# Retrospective Analysis Comparing Lung-RADS v2022 and British Thoracic Society Guidelines for Differentiating Lung Metastases from Primary Lung Cancer

**DOI:** 10.3390/biomedicines13010130

**Published:** 2025-01-08

**Authors:** Loredana Gabriela Stana, Alexandru Ovidiu Mederle, Claudiu Avram, Felix Bratosin, Paula Irina Barata

**Affiliations:** 1 Department I, Discipline of Anatomy and Embriology, Victor Babes University of Medicine and Pharmacy, Eftimie Murgu Square 2, 300041 Timisoara, Romania; loredana.stana@umft.ro; 2 Department of Surgery, Victor Babes University of Medicine and Pharmacy, 300041 Timisoara, Romania; mederle.ovidiu@umft.ro; 3 Doctoral School, Victor Babes University of Medicine and Pharmacy, 300041 Timisoara, Romania; 4 Department of Infectious Disease, Victor Babes University of Medicine and Pharmacy, 300041 Timisoara, Romania; felix.bratosin@umft.ro; 5 Center for Research and Innovation in Precision Medicine of Respiratory Diseases, Victor Babes University of Medicine and Pharmacy, 300041 Timisoara, Romania; barata.paula@student.uvvg.ro; 6 Department of Physiology, Faculty of Medicine, “Vasile Goldis” Western University of Arad, 310025 Arad, Romania

**Keywords:** Lung-RADS, British Thoracic Society guidelines, lung metastases, primary lung cancer, CT imaging, pulmonary nodules

## Abstract

**Background and Objectives**: The current study aimed to compare the effectiveness of the Lung Imaging Reporting and Data System (Lung-RADS) Version 2022 and the British Thoracic Society (BTS) guidelines in differentiating lung metastases from de novo primary lung cancer on CT scans in patients without prior cancer diagnosis. **Materials and Methods**: This retrospective study included 196 patients who underwent chest CT scans between 2015 and 2022 without a known history of cancer but with detected pulmonary nodules. CT images characterized nodules based on size, number, location, margins, attenuation, and growth patterns. Nodules were classified according to Lung-RADS Version 2022 and BTS guidelines. Statistical analyses compared the sensitivity and specificity of Lung-RADS and BTS guidelines in distinguishing metastases from primary lung cancer. Subgroup analyses were conducted based on nodule characteristics. **Results**: Of the 196 patients, 148 (75.5%) had de novo primary lung cancer, and 48 (24.5%) had lung metastases from occult primary tumors. Lung-RADS Version 2022 demonstrated higher specificity than BTS guidelines (87.2% vs. 72.3%, *p* < 0.001) while maintaining similar sensitivity (91.7% vs. 93.8%, *p* = 0.68) in differentiating lung metastases from primary lung cancer. Lung metastases were more likely to present with multiple nodules (81.3% vs. 18.2%, *p* < 0.001), lower lobe distribution (58.3% vs. 28.4%, *p* < 0.001), and smooth margins (70.8% vs. 20.3%, *p* < 0.001), whereas primary lung cancers were associated with solitary nodules, upper lobe location, and spiculated margins. **Conclusions**: Lung-RADS Version 2022 provides higher specificity than the BTS guidelines in differentiating lung metastases from primary lung cancer on CT scans in patients without prior cancer diagnosis. Recognizing characteristic imaging features can improve diagnostic accuracy and guide appropriate management.

## 1. Introduction

Lung cancer remains the leading cause of cancer-related mortality worldwide, accounting for approximately 18% of all cancer deaths [1]. The early detection and accurate characterization of pulmonary nodules are essential for improving patient outcomes and guiding appropriate management strategies [2]. While primary lung cancers are prevalent, the lungs are also a common site for metastases from extrapulmonary malignancies [3]. Differentiating between de novo primary lung cancer and lung metastases in patients without a prior cancer diagnosis poses a significant diagnostic challenge with critical clinical implications [4].

Computed tomography (CT) imaging plays a pivotal role in the detection and evaluation of pulmonary nodules [5,6]. Various guidelines have been developed to standardize reporting and management recommendations for pulmonary nodules, including the Lung Imaging Reporting and Data System (Lung-RADS) by the American College of Radiology [7] and the British Thoracic Society (BTS) guidelines [8]. Lung-RADS provides a structured framework for assessing lung cancer risk based on nodule characteristics, whereas the BTS guidelines offer detailed algorithms incorporating patient risk factors and nodule features.

Recent updates to Lung-RADS in Version 2022 have refined nodule classification criteria, potentially improving diagnostic accuracy and reducing false-positive rates [9]. However, the effectiveness of Lung-RADS Version 2022 compared to BTS guidelines in differentiating lung metastases from primary lung cancer in patients without prior cancer diagnoses has not been extensively studied. Given that management strategies differ significantly between primary lung cancer and metastatic disease, accurate differentiation is crucial [10].

Patients presenting with pulmonary nodules but no known malignancy require careful evaluation. Lung metastases in such patients may originate from occult primary tumors and often present with different imaging features compared to primary lung cancers [11]. Identifying specific imaging characteristics that distinguish metastases from primary tumors can prompt timely investigation for occult malignancies and guide appropriate management strategies [12].

Previous studies have focused on the utility of imaging features and guidelines in evaluating pulmonary nodules; however, there is limited research comparing Lung-RADS Version 2022 and BTS guidelines specifically in differentiating lung metastases from primary lung cancer in patients without prior cancer diagnoses [13]. Lung-RADS v2022 categorizes pulmonary nodules primarily based on size and morphological features: nodules are classified from Category 1 (negative) to Category 4X (suspicious) using size thresholds (e.g., 4–6 mm, 6–8 mm, >8 mm), attenuation patterns (solid, part-solid, ground-glass), and growth rates observed in follow-up scans. This system emphasizes standardized reporting to enhance consistency in lung cancer screening. In contrast, the BTS guidelines adopt a more holistic approach by incorporating patient-specific risk factors such as age, smoking history, and clinical symptoms alongside nodule characteristics like size, number (solitary vs. multiple), location (upper vs. lower lobes), and morphological features (smooth vs. spiculated margins). The BTS framework stratifies nodules into low-, intermediate-, or high-risk categories, providing tailored management recommendations based on the integrated assessment of these parameters.

Understanding the comparative effectiveness of these guidelines can aid radiologists and clinicians in making more accurate diagnoses and management decisions. Therefore, the purpose of this study was to compare the diagnostic performance of Lung-RADS Version 2022 and BTS guidelines in differentiating lung metastases from primary lung cancer on CT scans in patients without a prior cancer diagnosis. Additionally, we sought to identify key imaging features associated with lung metastases and evaluate their predictive value through subgroup analyses. By improving diagnostic accuracy, we aim to enhance patient care and optimize clinical outcomes.

## 2. Materials and Methods

### 2.1. Study Design and Patient Selection

The current research project was designed as a retrospective study with radiological interpretations from the Clinical Municipal Hospital from Timisoara, Romania, affiliated with the Victor Babes University of Medicine and Pharmacy from Timisoara, Romania, as well as from local private clinics. This observational study secured ethical approval from the Institutional Review Board, which adheres to the principles set forth in the Declaration of Helsinki. Additionally, this study complies with the EU Good Clinical Practice Directive (2005/28/EC) and the guidelines provided by the International Council for Harmonization of Technical Requirements for Pharmaceuticals for Human Use (ICH), which emphasize informed consent, scientific validity, and the safeguarding of participants’ health and rights.

The inclusion criteria for the study were patients aged 18 years or older with no prior history of any cancer diagnosis and the presence of one or more pulmonary nodules measuring at least 4 mm detected on CT scans. Exclusion criteria included any prior diagnosis of malignancy, CT scans of poor image quality or incomplete imaging data, nodules that exhibited benign features such as benign calcifications or fat density, and patients who did not undergo repeat imaging.

### 2.2. Imaging Protocol and Evaluation

All CT scans were conducted using standardized protocols on multidetector CT scanners, capturing images at full inspiration with slice thicknesses ranging from 1 to 2 mm. Intravenous contrast was used when clinically indicated. The assessment and characterization of pulmonary nodules included various features: size, measured in maximum diameter across axial, coronal, or sagittal planes; number, noting whether nodules were solitary or multiple; location, categorized as upper lobes, middle lobe/lingula, or lower lobes; margins, described as smooth, lobulated, or spiculated; attenuation, identified as solid, part-solid, or ground-glass; growth pattern, observed as stable, increased in size, or presenting new nodules on follow-up; and distribution pattern, noted as random, perilymphatic, or centrilobular. Additional observations included the presence of cavitation or calcification.

Primary lung cancer was defined as a new malignancy originating in the lung, typically presenting as solitary nodules in the upper lobes with spiculated margins. Metastatic lung cancer referred to cancers that spread to the lungs from other primary sites, usually appearing as multiple nodules in the lower lobes with smooth margins. The study used histopathological confirmation through biopsy or surgical resection as the gold standard to accurately differentiate between primary and metastatic lung cancers.

### 2.3. Application of Lung-RADS and BTS Guidelines

The application of Lung-RADS and BTS guidelines involved classifying each nodule using the Lung-RADS Version 2022 criteria, assigning categories from 1 (negative) to 4 (suspicious) based on size, attenuation, and growth patterns, alongside the BTS guidelines that stratify nodules into low, intermediate, or high risk considering nodule size, growth, and patient risk factors. Management recommendations for each nodule were documented as per both guidelines, with observations of features suggestive of metastases, such as multiple nodules and lower lobe distribution, also recorded.

Clinical data were gathered from electronic medical records, encompassing demographics (age, sex), smoking history (recorded in pack-years), presenting symptoms (such as cough, hemoptysis, and weight loss), and available laboratory tests (e.g., tumor markers). Follow-up data including biopsy results, surgical pathology, and clinical diagnoses were also collected. Patients were also looked-up for repeat imaging to establish final diagnoses, with histopathological confirmation obtained through biopsy or surgical resection when performed. Additional data were gathered in cases where a primary tumor was identified following the initial CT scan.

In this retrospective study, the diagnostic criteria for lung cancer were primarily based on computed tomography (CT) imaging characteristics of pulmonary nodules in patients without a prior cancer diagnosis. Specifically, nodules were evaluated for size (≥4 mm), number (solitary versus multiple), location (upper versus lower lobes), margins (smooth versus spiculated), attenuation (solid, part-solid, or ground-glass), and growth patterns (stable, increasing, or new nodules on follow-up). These features were systematically classified using both the Lung Imaging Reporting and Data System (Lung-RADS) Version 2022 and the British Thoracic Society (BTS) guidelines to differentiate de novo primary lung cancer from lung metastases. The study did not employ the TNM (Tumor, Node, Metastasis) staging system; instead, it focused on imaging-based classification criteria to assess and compare the diagnostic performance of Lung-RADS and BTS guidelines in identifying primary versus metastatic lung lesions.

### 2.4. Statistical Analysis

Statistical analyses were conducted using SPSS software version 26.0 (IBM Corp., Armonk, NY, USA). Continuous variables were expressed as mean ± standard deviation and compared using the Student’s *t*-test or Mann–Whitney U test as appropriate, while categorical variables were presented as counts and percentages and analyzed using chi-squared or Fisher’s exact tests. The sensitivity, specificity, positive predictive value (PPV), and negative predictive value (NPV) of the Lung-RADS and BTS guidelines in distinguishing metastases from primary lung cancer were calculated using the final diagnosis as the reference standard, with the McNemar test comparing the sensitivity and specificity between the two guidelines. Logistic regression analysis identified independent predictors of lung metastases, including variables significant in univariate analysis into the multivariate model, and reported odds ratios (ORs) with 95% confidence intervals (CIs). Subgroup analyses based on nodule size, number, location, margins, and patient smoking history assessed statistical significance with a *p*-value less than 0.05 deemed to be statistically significant. Nevertheless, future studies should examine whether differences in diagnostic classifications between Lung-RADS v2022 and BTS guidelines influence clinical decision making and alter treatment strategies for patients with pulmonary nodules.

## 3. Results

Table 1 presents the demographic and clinical characteristics of the study population. Patients with primary lung cancer were significantly older than those with lung metastases (mean age 66.2 vs. 58.9 years, *p* < 0.001). The majority of patients in both groups were male, but the difference was not statistically significant (*p* = 0.18). Smoking history, measured in pack-years, was significantly higher in patients with primary lung cancer compared to those with lung metastases (mean 34.5 vs. 11.2 pack-years, *p* < 0.001), highlighting the strong association between smoking and primary lung cancer. Symptoms were more commonly reported in the primary lung cancer group, with 74.3% experiencing symptoms compared to 41.7% in the metastases group (*p* < 0.001). Specifically, cough was significantly more prevalent in the primary lung cancer group (*p* = 0.002), whereas hemoptysis and weight loss showed no significant differences between groups.

Table 2 details the imaging characteristics of pulmonary nodules in both groups. Nodules in the primary lung cancer group were significantly larger than those in the metastases group (mean size 3.1 cm vs. 1.7 cm, *p* < 0.001). The majority of primary lung cancers presented as solitary nodules (81.8%), whereas lung metastases predominantly presented as multiple nodules (81.3%), with this difference being highly significant (*p* < 0.001). Regarding nodule location, primary lung cancers were more commonly located in the upper lobes (59.5%), while metastases were more frequently found in the lower lobes (58.3%), again showing a significant difference (*p* < 0.001). Nodule margins differed significantly between groups, with spiculated margins being more prevalent in primary lung cancers (67.6%) and smooth margins more common in metastases (70.8%, *p* < 0.001). There was no significant difference in nodule attenuation between groups (*p* = 0.36). Growth patterns on follow-up also varied, with primary lung cancers more likely to show increased size over time (70.3%) compared to metastases (29.2%, *p* = 0.001).

Table 3 compares the diagnostic performance of Lung-RADS Version 2022 and BTS guidelines in differentiating lung metastases from primary lung cancer. Lung-RADS demonstrated a high sensitivity of 91.7%, correctly identifying 44 of 48 patients with metastases, and a specificity of 87.2%, correctly identifying 129 of 148 patients with primary lung cancer. The BTS guidelines showed a similar sensitivity of 93.8% but a significantly lower specificity of 72.3% (*p* < 0.001). The higher specificity of Lung-RADS indicates a better ability to correctly identify patients without metastases, reducing false-positive rates. The positive predictive value (PPV) was higher for Lung-RADS (73.0% vs. 54.9%), suggesting that when Lung-RADS indicates metastases, there is a higher likelihood that the patient indeed has metastases. The negative predictive value (NPV) was high for both guidelines, indicating that when either guideline suggests no metastases, the patient is likely free of metastases. The overall accuracy was significantly higher for Lung-RADS (88.8% vs. 77.6%, *p* < 0.001).

Table 4 examines the relationship between nodule size and the likelihood of metastases. Among nodules ≤ 1 cm in size, a significantly higher proportion were lung metastases (37.5%) compared to primary lung cancers (13.5%, *p* < 0.001). Similarly, in the 1.1–2 cm size category, metastases accounted for 41.7% of nodules, while primary lung cancers comprised 25.7%. In contrast, nodules larger than 2 cm were predominantly primary lung cancers (60.8%), with only 20.8% being metastases. This trend suggests that smaller nodules are more likely to be metastases, whereas larger nodules are more commonly primary lung cancers.

Table 5 delves into the association between nodule number and location with the likelihood of metastases. The data reveal a significant difference in nodule number between the two groups (*p* < 0.001). The majority of primary lung cancers presented as solitary nodules (81.8%), whereas lung metastases predominantly presented as multiple nodules (81.3%). Regarding nodule location, primary lung cancers were more frequently located in the upper lobes (59.5%), while metastases were more commonly found in the lower lobes (58.3%, *p* < 0.001).

Table 6 explores the relationship between nodule margins and smoking history with the differentiation of metastases from primary lung cancer. A significant difference in nodule margins was observed between the two groups (*p* < 0.001). Smooth margins were more prevalent in lung metastases (70.8%), whereas spiculated margins were more common in primary lung cancers (67.6%). Regarding smoking history, a significant association was found (*p* < 0.001). Among patients with less than 20 pack-years of smoking history, 75% had lung metastases, while only 25% had primary lung cancer. Conversely, among those with a smoking history of 20 pack-years or more, 74.3% had primary lung cancer, and only 25% had metastases.

Table 7 presents the results of the multivariate logistic regression analysis identifying independent predictors of lung metastases. Multiple nodules emerged as the strongest predictor, with an adjusted odds ratio (OR) of 12.5 (*p* < 0.001), indicating that patients with multiple nodules are over 12 times more likely to have metastases than primary lung cancer. Smooth margins (OR = 7.0, *p* < 0.001) and lower lobe location (OR = 3.2, *p* = 0.003) were also significant independent predictors. Nodule size ≤ 2 cm was associated with a higher likelihood of metastases (OR = 2.5, *p* = 0.03). A smoking history of less than 20 pack-years was significantly associated with metastases (OR = 3.9, *p* = 0.001). Age was not a significant predictor in the multivariate model (*p* = 0.60).

## 4. Discussion

### 4.1. Important Findings and Literature Review

In this retrospective study of 196 patients without prior cancer diagnoses presenting with pulmonary nodules, we compared the effectiveness of Lung-RADS Version 2022 and BTS guidelines in differentiating lung metastases from primary lung cancer on CT scans. Our findings indicate that Lung-RADS Version 2022 provides higher specificity and overall accuracy than BTS guidelines, without compromising sensitivity.

The higher specificity of Lung-RADS Version 2022 suggests a reduced rate of false-positive diagnoses of metastases, potentially preventing unnecessary investigations and anxiety for patients. The similar sensitivity between the two guidelines indicates that both are effective at detecting metastases when present. These results support the use of Lung-RADS Version 2022 in clinical practice for more accurate differentiation between metastases and primary lung cancer.

Our subgroup analyses identified multiple nodules, lower lobe location, smooth margins, smaller nodule size, and minimal smoking history as significant predictors of lung metastases. These imaging features likely reflect the pathophysiology of metastatic disease, which tends to disseminate hematogenously, leading to multiple, well-circumscribed nodules preferentially distributed in the lower lobes due to higher blood flow [13]. In contrast, primary lung cancers often present as solitary, spiculated nodules in patients with significant smoking histories.

The multivariate logistic regression analysis confirmed that multiple nodules, smooth margins, lower lobe location, smaller nodule size, and minimal smoking history are independent predictors of metastases. These findings emphasize the importance of a comprehensive assessment of both imaging characteristics and clinical factors in the evaluation of pulmonary nodules.

In a similar manner, the study by Hendrix et al. [14] demonstrated the effectiveness of a deep learning-based AI system in the detection of benign and malignant pulmonary nodules in non-screening chest CT scans, achieving high sensitivity rates for detecting benign nodules, primary lung cancers, and metastases at 94.3%, 96.9%, and 92.0%, respectively. These rates were comparable to or higher than those achieved by a panel of radiologists, albeit with a slightly higher false positive per scan rate. Conversely, Li et al. [15] explored the utility of CT features and quantitative analysis in predicting the pathological classification of subsolid nodule lung adenocarcinoma, identifying significant differences in mean size, maximum diameter, and CT values among various adenocarcinoma stages. They developed logistic regression models that effectively predicted pathological classifications with high areas under the curve of 0.815 and 0.931.

Moreover, the study by the Canadian Agency for Drugs and Technologies in Health evaluated the diagnostic accuracy and cost-effectiveness of the Lung Imaging Reporting and Data System (Lung-RADS) compared to the Pan-Canadian Early Detection of Lung Cancer (PanCan) model [16]. Their findings indicate that the PanCan model might perform better at determining which lung nodules identified by low-dose CT are cancerous compared to Lung-RADS, based on variable quality evidence from six diagnostic test accuracy studies. However, other studies have suggested that both risk calculators have similar diagnostic accuracy. Economic evaluations have presented inconsistent results regarding the cost-effectiveness of the two models, influenced by the type of lung nodules applied in each study. On the other hand, the study by van Riel et al. [17] focused on observer variability in the assignment of Lung-RADS categories in lung cancer screening CTs, finding substantial interobserver agreement with a mean kappa of 0.67. Disagreements in Lung-RADS categorization affected patient management in a minor fraction of cases (8%), demonstrating the system’s robustness in clinical application despite some variability among readers.

Similarly, the study by Kaman Chung et al. [18] evaluated the accuracy of the Lung-RADS criteria for subsolid nodules (SSNs) in estimating malignancy risks, focusing on categories 2 and 4B within the National Lung Screening Trial (NLST). They found that the actual malignancy rates of 2.5% for category 2 SSNs and 23.9% for category 4B SSNs were slightly above and below, respectively, the expected probabilities defined by Lung-RADS (<1% for category 2 and >15% for category 4B). About one-third of benign lesions in both categories were transient, suggesting the potential value of integrating short-term follow-up to confirm persistence and reduce unnecessary procedures. Conversely, the comprehensive review by Gandhi et al. [19] highlighted the advancements of artificial intelligence (AI) in lung cancer screening, diagnosis, and management, showcasing AI’s capability to accurately detect and characterize lung nodules. AI algorithms, including machine learning and radiomics, have significantly enhanced the specificity and accuracy of lung cancer diagnostics, helping in the early detection and informed management of lung nodules.

Finally, a systematic review and meta-analysis by Yifei Mao et al. [20] assessed the performance of Lung-RADS across different target populations, highlighting substantial variability in its diagnostic accuracy based on population characteristics. They found that the pooled sensitivity of Lung-RADS version 1.0 was notably high at 0.96, with a specificity of 0.90. Interestingly, higher sensitivity was observed in high-risk populations compared to general populations, while non-Asian studies showed slightly better sensitivity than Asian studies. This study revealed that heterogeneity in sensitivity and specificity was influenced by the type of population (high-risk vs. general) and geographic region (Asia vs. non-Asia). Conversely, the study by Peng Huang et al. [21] explored a deep learning approach (DeepLR) to predicting lung cancer risk at follow-up screenings, showing impressive discrimination abilities with time-dependent AUC values of 0.968, 0.946, and 0.899 for 1-year, 2-year, and 3-year cancer diagnosis, respectively. DeepLR successfully identified a significant proportion of lung cancers early and was particularly effective in recognizing high-risk individuals, who also showed a higher risk of mortality.

The imaging characteristics of pulmonary nodules reveal distinct patterns that effectively differentiate primary lung cancer from metastatic lesions. Primary lung cancers typically present as larger, solitary nodules located predominantly in the upper lobes, often exhibiting spiculated margins which suggest a more aggressive and localized growth. In contrast, metastatic nodules are generally smaller, appear as multiple lesions, and are more frequently found in the lower lobes with smooth margins, indicating a hematogenous spread from an existing primary tumor. Additionally, metastatic nodules tend to remain stable over time, whereas primary lung cancers are more likely to show progressive growth. These differences in size, number, location, margins, and growth behavior are critical for radiologists and clinicians in accurately diagnosing and distinguishing between primary and metastatic lung tumors. By understanding these distinct imaging features, healthcare providers can enhance diagnostic precision, tailor appropriate management strategies, and ultimately improve patient outcomes. This analysis underscores the importance of detailed imaging assessments in the application of Lung-RADS v2022 and BTS guidelines, highlighting their roles in effectively categorizing and differentiating lung tumor types.

Nevertheless, it is important to acknowledge the absence of Positron Emission Tomography (PET) as a diagnostic tool, which remains a crucial technique in contemporary oncology. PET imaging significantly enhances the accuracy of differentiating pulmonary metastases from primary lung cancer by providing metabolic information that complements the anatomical details obtained from CT scans. The integration of PET could potentially improve the sensitivity and specificity of both Lung-RADS v2022 and BTS guidelines, offering a more comprehensive assessment of pulmonary nodules. Future research should incorporate PET alongside these guidelines to validate and possibly enhance their diagnostic performance, ensuring a more robust approach to identifying occult primary neoplasms and metastatic disease.

### 4.2. Study Limitations

This study has several limitations. First, as a retrospective analysis, it is subject to selection bias and may not be generalizable to all populations. This study relied on the availability and accuracy of medical records and imaging data. Second, histopathological confirmation was not available for all patients; some diagnoses were based on clinical and radiological follow-up, which may introduce diagnostic uncertainty. Third, we did not evaluate the impact of advanced imaging modalities such as PET-CT, which could provide additional diagnostic information. Additionally, the sample size, while adequate, limits the power of subgroup analyses, particularly for less common variables. Future prospective studies with larger cohorts and standardized imaging protocols are warranted to validate these findings. Including advanced imaging techniques and molecular markers may further enhance diagnostic accuracy and improve patient outcomes.

## 5. Conclusions

Despite the promising findings that Lung-RADS Version 2022 may offer higher specificity and overall accuracy compared to BTS guidelines in differentiating lung metastases from primary lung cancer in patients without prior cancer diagnoses, these results should be interpreted with caution due to several study limitations. The retrospective nature of the analysis introduces potential selection bias and limits generalizability, while the reliance on clinical and radiological follow-up for some diagnoses may introduce diagnostic uncertainty. Additionally, the absence of advanced imaging modalities such as PET-CT and the relatively limited sample size constrain the strength of our conclusions and the power of subgroup analyses. Therefore, while Lung-RADS Version 2022 demonstrates potential as a valuable tool in the assessment of pulmonary nodules, further prospective studies with larger cohorts, standardized imaging protocols, and the inclusion of advanced diagnostic techniques are necessary to validate these findings and fully establish the comparative effectiveness of Lung-RADS and BTS guidelines. Future research should also explore the impact of diagnostic variations on therapeutic decisions and treatment outcomes to enhance patient care.

## Figures and Tables

**Table 1:** Patient demographics and clinical characteristics.

Characteristic	Primary Lung Cancer (n = 148)	Lung Metastases (n = 48)	*p*-Value
Age (years)	66.2 ± 9.7	58.9 ± 10.5	<0.001
Male sex	96 (64.9%)	26 (54.2%)	0.18
Smoking history (pack-years)	34.5 ± 16.0	11.2 ± 9.5	<0.001
Symptoms present	110 (74.3%)	20 (41.7%)	<0.001
Cough	68 (45.9%)	10 (20.8%)	0.002
Hemoptysis	24 (16.2%)	4 (8.3%)	0.18
Weight loss	18 (12.2%)	6 (12.5%)	0.96
Histological subtypes			
Adenocarcinoma	88 (59.5%)	17 (35.4%)	<0.001
Squamous cell carcinoma	40 (27.1%)	6 (12.6%)	0.002
Large cell carcinoma	20 (13.5%)	3 (6.2%)	0.08
Primary tumor location			
Upper lobe	91 (61.5%)	16 (33.3%)	<0.001
Middle lobe/lingula	19 (12.8%)	8 (16.7%)	0.34
Lower lobe	38 (25.7%)	24 (50.0%)	<0.001

**Table 2:** Imaging characteristics of pulmonary nodules.

Characteristic	Primary Lung Cancer (n = 148)	Lung Metastases (n = 48)	*p*-Value
Nodule size (cm)	3.1 ± 1.5	1.7 ± 0.8	<0.001
Nodule number			<0.001
Solitary	121 (81.8%)	9 (18.8%)	
Multiple	27 (18.2%)	39 (81.3%)	
Nodule location		<0.001	0.01
Upper lobes	88 (59.5%)	14 (29.2%)	
Middle lobe/lingula	18 (12.2%)	6 (12.5%)	
Lower lobes	42 (28.4%)	28 (58.3%)	
Nodule margins		<0.001	<0.001
Smooth	30 (20.3%)	34 (70.8%)	
Lobulated	18 (12.2%)	6 (12.5%)	
Spiculated	100 (67.6%)	8 (16.7%)	
Attenuation			0.36
Solid	132 (89.2%)	44 (91.7%)	
Part solid	10 (6.8%)	2 (4.2%)	
Ground glass	6 (4.1%)	2 (4.2%)	
Growth on follow-up		0.001	
Stable	44 (29.7%)	34 (70.8%)	
Increased size	104 (70.3%)	14 (29.2%)	

**Table 3:** Diagnostic performance of Lung-RADS Version 2022 and BTS guidelines.

Metric	Lung-RADS Version 2022	BTS Guidelines	Total Nodules
Differentiating Metastases			<0.001
Sensitivity (%)	91.7	93.8	0.68
Specificity (%)	87.2	72.3	<0.001
Positive Predictive Value (%)	73	54.9	
Negative Predictive Value (%)	96.2	94.9	
Overall Accuracy (%)	88.8	77.6	<0.001

**Table 4:** Nodule size and likelihood of metastases.

Nodule Size Category	Primary Lung Cancer (n = 148)	Lung Metastases (n = 48)	*p*-Value
≤1 cm	20 (13.5%)	18 (37.5%)	<0.001
1.1–2 cm	38 (25.7%)	20 (41.7%)	
>2 cm	90 (60.8%)	10 (20.8%)	

**Table 5:** Nodule number and location in differentiating metastases.

Characteristic	Primary Lung Cancer (n = 148)	Lung Metastases (n = 48)	*p*-Value
Nodule Number		<0.001	<0.001
Solitary	121 (81.8%)	9 (18.8%)	
Multiple	27 (18.2%)	39 (81.3%)	
Nodule Location		<0.001	
Upper lobes	88 (59.5%)	14 (29.2%)	
Middle lobe/lingula	18 (12.2%)	6 (12.5%)	
Lower lobes	42 (28.4%)	28 (58.3%)	

**Table 6:** Nodule margins and smoking history in differentiating metastases.

Characteristic	Primary Lung Cancer (n = 148)	Lung Metastases (n = 48)	*p*-Value
Nodule Margins			<0.001
Smooth	30 (20.3%)	34 (70.8%)	
Lobulated	18 (12.2%)	6 (12.5%)	
Spiculated	100 (67.6%)	8 (16.7%)	
Smoking History			<0.001
<20 pack-years	38 (25.7%)	36 (75.0%)	
≥20 pack-years	110 (74.3%)	12 (25.0%)	

**Table 7:** Multivariate logistic regression analysis for predictors of lung metastases.

Variable	Adjusted OR (95% CI)	*p*-Value
Multiple nodules	12.5 (5.8–27.0)	<0.001
Lower lobe location	3.2 (1.5–6.9)	0.003
Smooth margins	7.0 (3.1–15.8)	<0.001
Nodule size ≤ 2 cm	2.5 (1.1–5.6)	0.03
Smoking history < 20 pack-years	3.9 (1.8–8.5)	0.001
Age	1.2 (0.6–2.2)	0.6

## Data Availability

The data presented in this study are available on request from the corresponding author. The data are not publicly available due to due to legal and ethical reasons.
